# ANKRD22 contributes to NF-κB-associated inflammatory responses in alveolar type II epithelial cells during PM_2.5_-triggered airway inflammation

**DOI:** 10.3389/fimmu.2026.1894350

**Published:** 2026-09-16

**Authors:** Yan Jin, Yun Zhao, Shihui Wei, Xingyu Chen, Ke Zhu, Juan Xiong, Rongbin Qi, Wen Zhang, Tianwen Lai, Chao Cao

**Affiliations:** 1 Department of Respiratory and Critical Medicine, Key Laboratory of Respiratory Disease of Ningbo, The First Affiliated Hospital of Ningbo University, Ningbo, China; 2 Department of Respiratory and Critical Care Medicine, Taizhou Municipal Hospital (Taizhou University Affiliated Municipal Hospital), School of Medicine, Taizhou University, Taizhou, China; 3 Dongguan Key Laboratory of Immune Inflammation and Metabolism, The First Dongguan Affiliated Hospital, Guangdong Medical University, Dongguan, China

**Keywords:** airway inflammation, alveolar type II epithelial cells, ANKRD22, NF-κB, PM2.5

## Abstract

**Background:**

PM_2.5_ causes multifaceted detrimental effects on human health. Alveolar type II epithelial (AT-II) cells serve critical function in PM_2.5_-induced airway inflammation. However, the mechanism remains unclear.

**Methods:**

ANKRD22 expression in AT-II cells was assessed in a PM_2.5_-induced mouse airway inflammation model using immunofluorescence, qRT-PCR and Western blot. AT-II cell-specific ANKRD22 knockout (AT-II-*ANKRD22^△/△^*) mice were generated and exposed to PM_2.5_. Inflammation was evaluated by BALF leukocyte and neutrophil counts, lung H&E staining, and qRT-PCR/ELISA for IL-6 and CXCL1. *In vitro*, ANKRD22 knockdown in A549 cells was performed prior to PM_2.5_ stimulation, and cytokine levels (*IL-6, IL-8, IL-1β, CXCL1, CXCL2*) were measured by qRT-PCR and ELISA. RNA sequencing was used to explore the regulatory mechanisms of ANKRD22 in AT-II cells during PM_2.5_-induced airway inflammation.

**Results:**

Our study demonstrated that PM_2.5_ promoted ANKRD22 expression in AT-II cells both *in vivo* and *in vitro*. PM_2.5_-induced inflammation was alleviated in AT-II- *ANKRD22^△/△^* mice. The expression of *IL6*, *IL8*, *IL1B*, *CXCL1*, and *CXCL2* was downregulated by *ANKRD22* siRNA in A549 cells. Mechanistically, PM_2.5_ exposure significantly enhanced the protein expression of P50 and the phosphorylation of IKKβ, IκBα and RELA. Moreover, these PM_2.5_-induced changes were markedly attenuated following ANKRD22 deficiency. Similarly, PM_2.5_ exposure induced significant upregulation of p52 protein levels and RELB phosphorylation, which were substantially reduced by ANKRD22 knockdown.

**Conclusion:**

In conclusion, PM_2.5_ stimulation promoted the ANKRD22 expression in AT-II cells, thereby contributing to NF-κB-associated inflammatory responses during PM2.5-induced airway inflammation.

## 1 Introduction

Particulate matter smaller than 2.5 µm in size (PM_2.5_) plays a significant role in the initiation and progression of airway inflammation. Epidemiological and experimental studies have consistently established a strong association between PM_2.5_ exposure levels and the incidence of respiratory inflammatory diseases (1–4). Despite recent advances in PM_2.5_ control measures, the adverse biological effects of PM_2.5_ remain important contributors to the pathogenesis of airway inflammation (1). Asthma guidelines recognize PM_2.5_ as a major contributor to both the pathogenesis and clinical deterioration of refractory asthma (5). Prolonged exposure to PM_2.5_ has been associated with substantial declines in lung function, with an estimated 11.6% increase in mortality risk per 11 µg/m³ increment in PM_2.5_ concentration (4). Moreover, global epidemiological data on lung cancer indicate that the primary mechanism by which PM_2.5_ promotes lung carcinogenesis is not through the induction of novel DNA mutations, but rather through persistent inflammation (6). The World Health Organization has cautioned that once PM_2.5_ concentrations exceed certain thresholds, mortality risk increases significantly. Notably, even short-term exposure to PM_2.5_ can cause measurable damage to the body.

Pulmonary epithelial cells serve as one of the primary targets of PM_2.5_ upon inhalation. As both structural components of the respiratory system and key immunomodulators, these cells play a pivotal role in the pathogenesis of airway inflammatory diseases (7–9). Our previous studies confirmed the critical regulatory function of pulmonary epithelial cells in PM_2.5_-induced airway inflammation (10). However, the precise mechanisms by which these cells mediate PM_2.5_-induced airway inflammation remain unclear.

ANKRD22 (Ankyrin Repeat Domain-Containing Protein 22) is a member of the ankyrin repeat protein family, characterized by its signature ankyrin repeat motifs (11, 12). ANKRD22 expression exhibits tissue specificity and pathology-dependent patterns (13). It is predominantly found in gastrointestinal epithelium, activated monocytes/macrophages, and various tumor cells, including lung carcinoma (14–17). Accumulating evidence has implicated ANKRD22 in multiple malignancies, encompassing lung cancer, gastrointestinal tumors, breast cancer, and ovarian carcinoma (14, 15, 17–21). Notably, ANKRD22 exerts tumor-specific regulatory functions that vary significantly across cancer types and, in some cases, even demonstrates dichotomous roles within the same tumor category.

Recent studies have revealed ANKRD22 as an important regulatory molecule in inflammatory diseases. In gastric mucosal injury, ANKRD22 promotes gastritis progression. Its deletion in gastric epithelial cells enhances leucine-rich repeat-containing G-protein-coupled receptor 5-positive (Lgr5+) cell proliferation and mucosal repair. Conversely, ANKRD22 knockout in macrophages suppresses their activation, thereby alleviating gastric inflammation, suggesting ANKRD22 as a potential therapeutic target for gastric mucosal damage (15). Intriguingly, ANKRD22 exerts divergent functions in psoriasis pathogenesis. In imiquimod (IMQ)-induced psoriasiform mouse model, ANKRD22-deficient mice exhibited aggravated disease progression, an effect primarily due to dendritic cell-specific ANKRD22 deletion which selectively upregulated IL-23 production without affecting other cytokines. Mechanistically, ANKRD22 binds NIK to promote its ubiquitination and degradation, curbing non-canonical NF-κB activation; its loss sustains this pathway, exacerbating IL-23 production and psoriasis, positioning ANKRD22 as a potential therapeutic target (22). Despite these findings, the role of ANKRD22 in airway inflammation remains undefined, with no mechanistic or phenotypic studies reported to date.

We demonstrate that PM_2.5_ induced the expression of ANKRD22 in AT-II cells. Through establishing transgenic mice with specific knockout of ANKRD22 in AT-II cells and genetic approaches to inhibit ANKRD22 *in vitro*, we demonstrate that ANKRD22 deletion attenuates airway inflammation induced by intratracheal PM_2.5_ instillation. This protective effect was accompanied by reduced NF-κB-associated inflammatory responses and changes in both canonical and non-canonical NF-κB signaling components.

## 2 Materials and methods

### 2.1 Animals and cell lines

Our study has received approval for research ethics from the Animal Care and Use Committee at Ningbo University (NBU20220229). Wild-type C57BL/6 mice (6–8 weeks, male) were purchased from Shanghai SLAC Laboratory Animal Co., Ltd (Shanghai, China) and maintained in the Laboratory animal center of Ningbo University. *ANKRD22^flox/flox^* mice (C57BL/6 background) and *Sftpc-MerCreMer* transgenic mice were provided by Cyagen Biosciences Inc (Shanghai, China). The *Sftpc* encodes pulmonary surfactant protein C (SP-C). *Sftpc-MerCreMer- ANKRD22^flox/flox^* mice were used in animal experiments, and their littermate controls (*ANKRD22*^flox/flox^) were used as controls. Tail biopsies were collected from the offspring, and genomic DNA was extracted for genotyping. The following primer sequences were used: *Sftpc* (forward: 5’-AGGTGAGCAACAGTACCTTTCAG-3’, reverse: 5’-CCAAAAGACGGCAATATGGTGGAA-3’), *MerCreMer* (forward:5’-AATGCAAGAACGTGGTACCCCTC-3’, reverse: 5’-GGTTCTTGCGAACCTCATCACTC-3’), *ANKRD22^flox/flox^* (forward: 5’- ATCCTTTTAAACAGTGTTCCACGG-3’, reverse: 5’- GATTCTAAATA CAAGGCACTGCGA-3’). To induce the activation of *MerCreMer* recombinase, 8-12-week-old mice received intraperitoneal injections of tamoxifen solution (diluted in pure corn oil to a concentration of 20 mg/mL). Briefly, these mice were received intraperitoneal injection of tamoxifen (0.2 mg/g) every other day for a total 4 injections (23). Under tamoxifen induction, *MerCreMer* recombinase mediated the excision of 4–6 exon in the Ankrd22 gene (encoding ANKRD22), resulting in AT-II cell-specific ANKRD22 knockout (designated as *Sftpc-MerCreMer-ANKRD22^flox/flox^*-Tamo+, abbreviated as AT-II-*ANKRD22^△/△^*). To exclude potential effects of Cre recombinase or tamoxifen itself, Cre-negative littermate controls were also treated with tamoxifen. After the final injection, mice were allowed to rest for 5–7 days in advance of subsequent experiment. This work has received approval for research ethics from the Animal Care and Use Committee at Ningbo University (NBU20220229) and a proof/certificate of approval is available upon request.

A549 cell line used in our study was purchased from the Cell Bank of Chinese Academy of Sciences (Shanghai). Cells were maintained in complete DMEM/F12 medium (DMEM/F12+/+) containing 10% FBS (F101-01, Vazyme, China) and penicillin-streptomycin (C0222, Beyotime, China) at 37 °C with 5% CO_2_.

### 2.2 PM_2.5_ preparation and animal experiments

PM_2.5_ (1649B, NIST, USA) was suspended in sterile normal saline (NS), fully sonicated, and diluted to a concentration of 10 mg/mL. Sex- and age- matched mice were used in our study. To create a PM_2.5_-induced airway inflammation model in mice, 100 μg PM_2.5_ (in 50 μL saline) was intratracheally instilled for 4 consecutive days, and the control mice were treated with NS (10, 24).

### 2.3 BALF collection and analysis

Mice were sacrificed 24 h after the final PM_2.5_ or NS administration. The right main bronchus was ligated with a silk suture. and the right lung was lavaged three times with 0.4 mL of sterile PBS via an intratracheal cannula. The recovered bronchoalveolar lavage fluid (BALF) was pooled for each mouse. An aliquot of the BALF was used for total cell counting using an automated cell counter. The remaining BALF was centrifuged (4°C, 3,000 rpm, 10 min), and the supernatant was collected and stored at -80°C for further analysis. The cell pellet was resuspended in PBS, followed by cytospin (cytofuge2, StatSpin, USA). When the cell smear was dried, Giemsa staining (BA4017, Baso, China) was performed. For differential cell counting, 200 cells were counted for each smear. Neutrophils, eosinophils, lymphocytes and macrophages were classified. BALF differential cell counting was performed blinded to group allocation.

### 2.4 Lung tissue histopathological examination

Following BALF collection, the right lung was fixed in 4% paraformaldehyde (G1101, Servicebio, China), embedded in paraffin, and sectioned. Subsequently, hematoxylin-eosin (HE) staining was performed. The severity of lung inflammation was assessed based on neutrophil infiltration, alveolar wall thickness, and alveolar hemorrhage. The scoring system is as follows: Score 0: no detectable injury; Score 1: ≤ 25% of the field affected; Score 2: 26%-50% affected; Score 3: 51%-75% affected; Score 4: > 75% affected. Histological scoring was performed blinded to group allocation.

### 2.5 Immunofluorescence analysis of ANKRD22 in murine AT-II cells

Paraffin-embedded sections were deparaffinized, rehydrated, and subjected to antigen retrieval. After three washes with PBS (5 min each), hydrophobic barriers were drawn around the tissue using a hydrophobic pen, followed by blocking with 3% BSA for 30 min. Subsequently, the sections were incubated with a mixture of the SP-C and ANKRD22 primary antibodies (diluted according to the manufacturer’s instructions) at 4 °C overnight in a humidified chamber. After washing with PBS, the sections were incubated with a mixture of fluorescent secondary antibodies —Alexa Fluor 488-conjugated goat anti-rabbit IgG (A-11008, Invitrogen, USA) and CY3-conjugated goat anti-mouse IgG (ab97035, Abcam, UK)—for 50 min at room temperature in the dark. Afterward, nuclei were counterstained with DAPI for 5 min, an anti-fade mounting medium was added before coverslipping. Finally, images were acquired using a fluorescence microscope.

### 2.6 RNA extraction and qRT-PCR

Left lung tissues were dissected, transferred to pre-chilled bead mill tubes containing 1 mL RNAex Pro reagent (AG21101, AG, China), and homogenized at 60 Hz for 2 min. Total RNA was extracted according to the manufacturer’s instructions. Evo M-MLV RT-PCR Kit (AG11603, AG, China) was used for RNA reverse transcription. The expression of mouse *Il6*, *Cxcl1*, *Cxcl2*, *Il1b*, and human *IL6*, *IL8*, *CXCL1*, *CXCL2*, *IL1B*, *ANKRD22* was measured by qRT-PCR using SYBR Green Master Mix (AG11759, AG Biotechnology) on a real-time PCR system (LightCycler 96, Roche, Switzerland). The expression of genes was normalized to *Actin* (Mus) or *ACTIN* (Homo). All primers were synthesized by Sangon Biotech. The following primer sequences were used (Sangon Biotech): *Actin* (Forward: 5’-GGCTGTATTCCCCTCCATCG-3’, Reverse: 5’-CCAGTTGGTAACAATGCCATGT-3’), *Il6* (Forward: 5’-CTGCAAGAGACTTCCA TCCAG-3’, Reverse: 5’-AGTGGTATAGACAGGTCTGTTGG-3’), *Cxcl1* (Forward: 5’-CTGGGATTCAC CTCAAGAACATC-3’, Reverse: 5’-CAGGGTCAAGGCAAGC CTC-3’), *Cxcl2* (Forward: 5’-TGTCCCTCAACGGAAGAACC-3’, Reverse: 5’-CTCAGACAGCGAGGCACATC-3’), *Il1b* (Forward: 5’-ATGCCACCTTTTGACAG TGATG-3’, Reverse: 5’-AGCTTCTCCACAGCCACAAT-3’), *ACTIN* (Forward: 5’-CATGTACGTTGCTA TCCAGGC-3’, Reverse: 5’-CTCCTTAATGTCACGCACGAT-3’), *IL6* (Forward: 5’-ACTCACCTCTTCAGAACGAATTG-3’, Reverse: 5’-CCATCTTTGGAAGGTTCAGGTTG-3’). *IL8* (Forward: 5’-CACCATGACTTCCAA GCTGGC-3’, Reverse: 5’-TTATGAATTCTCAGCCCTCTTC-3’). *CXCL1* (Forward: 5’-TCTGGCTTAGAACAAAGGGGC-3’, Reverse: 5’-TAAAGGTAGCCCTTGTTT CCCC-3’), *CXCL2* (Forward: 5’-AGATCAATGTGACGGCAGGG-3’, Reverse: 5’-TCTCTGCTCTAACACAGAGGGA-3’), *IL1B* (Forward: 5’-ATGATGGCTTATTAC AGTGGCAA-3’, Reverse: 5’-GTCGGAGATTCGTAGCTGGA-3’) and *ANKRD22* (Forward: 5’-GCTCTTGTACGAATGCTACTTGA-3’, Reverse: 5’-CTCCGTGCAAT ATCCAGTGAG-3’).

### 2.7 Cell culture and siRNA transfection

A549 cells, a human AT-II cell line, were used for *in vitro* experiments. The cells were obtained from the Cell Bank of the Chinese Academy of Sciences (Shanghai, China). DMEM/F12 medium containing 10% FBS (F101-01, Vazyme, China) and penicillin-streptomycin (C0222, Beyotime, China), abbreviated as DMEM/F12(+/+) was used for A549 cell culture. The ANKRD22 siRNAs (designated as 334, 459, and 640, respectively) and NC siRNA used in our study were designed and synthesized by Shanghai Jima Gene Co., LTD. The siRNA sequences were as follows: ANKRD22 siRNA-334 (Forward: 5’-CGUUCAAGAUGGCUUUAA UTT-3’, Reverse: 5’- AUUAAAGCCAUCUUGAACGTT-3’), ANKRD22 siRNA-459 (Forward: 5’- CCUGCUUGCAUUAUGCUGUTT -3’, Reverse: 5’- ACAGCAUAAU GCAAGCAGGTT -3’), ANKRD22 siRNA-640 (Forward: 5’-CUGUACCGCAUUA CAUUAUTT-3’, Reverse: 5’-AUAAUGUAAUGCGGUACAGTT-3’) and NC siRNA (Forward: 5’-UUCUCCGAACGUGUCACGUTT-3’, Reverse: 5’-ACGUGACACG UUCGGAGAATT-3’). A549 cells were seeded into a 6-well plate (4 × 10^5^ cells/well). After cell culture for 16–18 h at approximately 80% confluence, the culture medium was replaced and siRNA transfection was carried out using GenMute transfection reagents (SLE100568, Signagen, China) following manufacturer’s protocol. The knockdown efficiency of each siRNA was evaluated 48 h post-transfection by quantitative real-time PCR (qRT-PCR) and Western blotting to identify the most efficient ANKRD22 siRNA. Following overnight siRNA transfection, the medium was replaced, and cells were stimulated with PM2.5 (200 μg/mL) or NS for 24 h.

### 2.8 Western blot assay

Cells were lysed in RIPA lysis buffer (P0013B, Beyotime, China) supplemented with a protease and phosphatase inhibitor cocktail, followed by ultrasonic disruption. The lysate was then centrifuged (4 °C, 13,000 rpm, 20 min), the supernatants were collected as total protein extracts and protein concentrations were measured by the Pierce BCA protein assay kit (23225, Thermo Fisher Scientific, USA). Equal amounts of protein were then mixed with loading buffer, boiled at 95 °C for 8 min, separated by SDS-PAGE, and transferred onto PVDF membranes. Afterwards, the membrane was blocked with 5% BSA and incubated with primary antibody at 4 °C overnight, followed by incubation with HRP-conjugated secondary antibodies for 1 h at room temperature. Protein bands were visualized using enhanced chemiluminescence (ECL) and quantified using ImageJ software. Actin served as an internal control. Primary antibodies used in our study were listed as follows: Actin (PA1-46296, Invitrogen, USA), ANKRD22 (NBP1-81167, Novus, USA), P50 (14220-1-AP, proteintech, USA), RELA (10745-1-AP, proteintech, USA), p-RELA (93H1, CST, USA), Iκκβ (A19606, ABclonal, China), p-IKKα/β (#2697, CST, USA), IκBα (A19714, ABclonal, China), p-IκBα (AP0707, ABclonal, China), RELB (25027-1-AP, proteintech, USA), p-RELB (#5025, CST, USA), P52 (10409-2-AP, ABclonal, China). Secondary antibodies used in our study were as follows: Goat Anti-Rabbit IgG (H+L) HRP (S0001, Affinity, China) and Goat Anti-Mouse IgG (H+L) HRP (S0002, Affinity, China).

### 2.9 ELISA

Left lung tissues of mouse were rinsed in clean PBS solution to remove blood debris, blotted dry with sterile, nuclease-free filter paper, and weighed. Then the tissues were transferred to pre-chilled bead mill tubes containing 1 mL of RIPA lysis buffer supplemented with protease and phosphatase inhibitors and homogenized at 60 Hz for 2 min. Subsequently, the homogenates were centrifuged at 13,000 rpm at 4 °C for 15 min. After centrifugation, the supernatants were collected as total protein extracts. The protein concentrations were measured using the Pierce BCA Protein Assay Kit (23225, Thermo scientific, China). The levels of IL-6 (RK00008, ABclonal, China) and CXCL1 (RK00038, ABclonal, China) in lung tissues were measured by ELISA according to the manufacturer’s instructions. *In vitro* experiment, the culture supernatants from A549 cells were collected, and the protein level of IL-6 (RK00004, ABclonal, China) and IL-8 (RK00011, ABclonal, China) were measured by ELISA.

### 2.10 RNA-Seq

A549 cells were transfected with ANKRD22 siRNA or NC siRNA followed by PM_2.5_ exposure. After 48 h of transfection, cells were harvested for total RNA extraction. The RNA samples were subsequently sent to BGI Genomics Co., Ltd for RNA sequencing.

RNA-Seq data were analyzed from four groups: NC siRNA-NS, NC siRNA-PM_2.5_, ANKRD22 siRNA-NS, and ANKRD22 siRNA-PM_2.5_. The raw sequencing data were filtered using SOAPnuke (v2.2.1) to obtain clean reads. Reads containing adapters, reads with more than 5% unknown (N) bases, and low-quality reads (defined as reads in which bases with a quality score below Q15 accounted for more than 20% of the total read length) were discarded. Clean reads were aligned to the reference gene set using Bowtie2 (v2.4.5). Gene expression levels were quantified using RSEM (v1.3.1). Principal component analysis (PCA) and Pearson correlation analyses based on variance-stabilizing transformation (VST)-transformed expression values were performed to evaluate sample clustering and biological reproducibility. Differential expression analysis was performed using DESeq2 (v1.34.0). Differentially expressed genes (DEGs) were identified by integrating two differential expression analyses: (1) PM_2.5_-responsive genes were screened by comparing NC siRNA-PM_2.5_ versus NC siRNA-NS; (2) ANKRD22-dependent genes were identified by comparing ANKRD22 siRNA-PM_2.5_ versus NC siRNA-PM_2.5_. Genes significantly altered by PM_2.5_ and reversed after ANKRD22 knockdown were considered ANKRD22-regulated PM_2.5_-responsive genes. DEGs were defined as |log_2_FC| (log_2_ Fold Change) ≥ 1 and an adjusted *P*-value < 0.05. Heatmaps of gene expression profiles were generated using the pheatmap package (v1.0.12). Subsequent data processing, visualization, and functional annotation were performed using the Dr. Tom multi-omics data mining system (https://biosys.bgi.com). To investigate the biological functions associated with DEGs, Gene Ontology (GO; http://www.geneontology.org/) and Kyoto Encyclopedia of Genes and Genomes (KEGG; https://www.kegg.jp/) enrichment analyses were performed using Phyper based on the hypergeometric test. Terms with an adjusted *P*-value < 0.05 were considered significantly enriched. Gene Set Enrichment Analysis (GSEA) was performed using Hallmark gene sets from the Molecular Signatures Database (MSigDB). Genes were ranked according to the ranking metric calculated as sign(log_2_FC) × −log_10_(*P*-value), which integrates both the direction and statistical significance of differential expression changes. Pathway significance was evaluated based on normalized enrichment score (NES), nominal *P*-value, and FDR. The raw RNA-Seq data have been deposited in the Mendeley Data repository (doi: 10.17632/xgf3swc26z.1).

### 2.11 Statistical analysis

All data were presented as means ± SEM. Data are representative of three biological replicates with similar results. For *in vitro* experiments, the sample size (n) represents the number of cell samples per group. For *in vivo* experiments, the sample size (n) represents the number of mice per group. Statistical analysis was performed with GraphPad Prism 8.0 software (GraphPad Software, Inc., San Diego, CA). The Differences between two groups were assessed using t-test, and one-way ANOVA followed by Bonferroni’s *post hoc* test was conducted for multiple groups. Statistical significance was indicated as follows: *P < 0.05, **P < 0.01, ***P < 0.001. All methods are reported in accordance with ARRIVE guidelines (https://arriveguidelines.org). All methods were carried out in accordance with relevant guidelines and regulations.

## 3 Results

### 3.1 PM_2.5_ upregulates ANKRD22 expression in AT-II cells *in vivo* and *in vitro*

We previously showed that intratracheal instillation of PM2.5 induces airway inflammation in mice (10). To examine the expression of ANKRD22 in AT-II cells, we first established a PM_2.5_-induced airway inflammation model in wild-type C57BL/6 mice. Given that AT-II cells are the primary source of pulmonary surfactant, and that surfactant-associated protein C (SP-C) is encoded by the *Sftpc* gene and serves as a specific marker for AT-II cells, we performed co-immunofluorescence staining for SP-C and ANKRD22. In PM_2.5_-exposed mice, we observed a marked increase in ANKRD22 signal within AT-II cells compared with saline-treated controls (**Figure 1A**). We next extended these observations to A549 cells, a widely used alveolar epithelial cell line. A549 cells were treated with PM_2.5_ at graded concentrations (0, 50, 100, and 200 μg/mL) and for varying time intervals (0, 6, 12, and 24 h). Both ANKRD22 mRNA and protein levels rose progressively with increasing PM_2.5_ concentrations, reaching statistical significance (*P* < 0.05) at the highest dose (200 μg/mL) (**Figures 1B–D**). Under 200 μg/mL PM_2.5_ exposure, ANKRD22 expression exhibited gradual upregulation over time, peaking at 24 h (**Figures 1E–G**). Collectively, these results demonstrate that PM_2.5_ exposure drives ANKRD22 upregulation in murine AT-II cells *in vivo* and induces a dose- and time-dependent upregulation of ANKRD22 expression in A549 cells *in vitro*.

**Figure 1:**
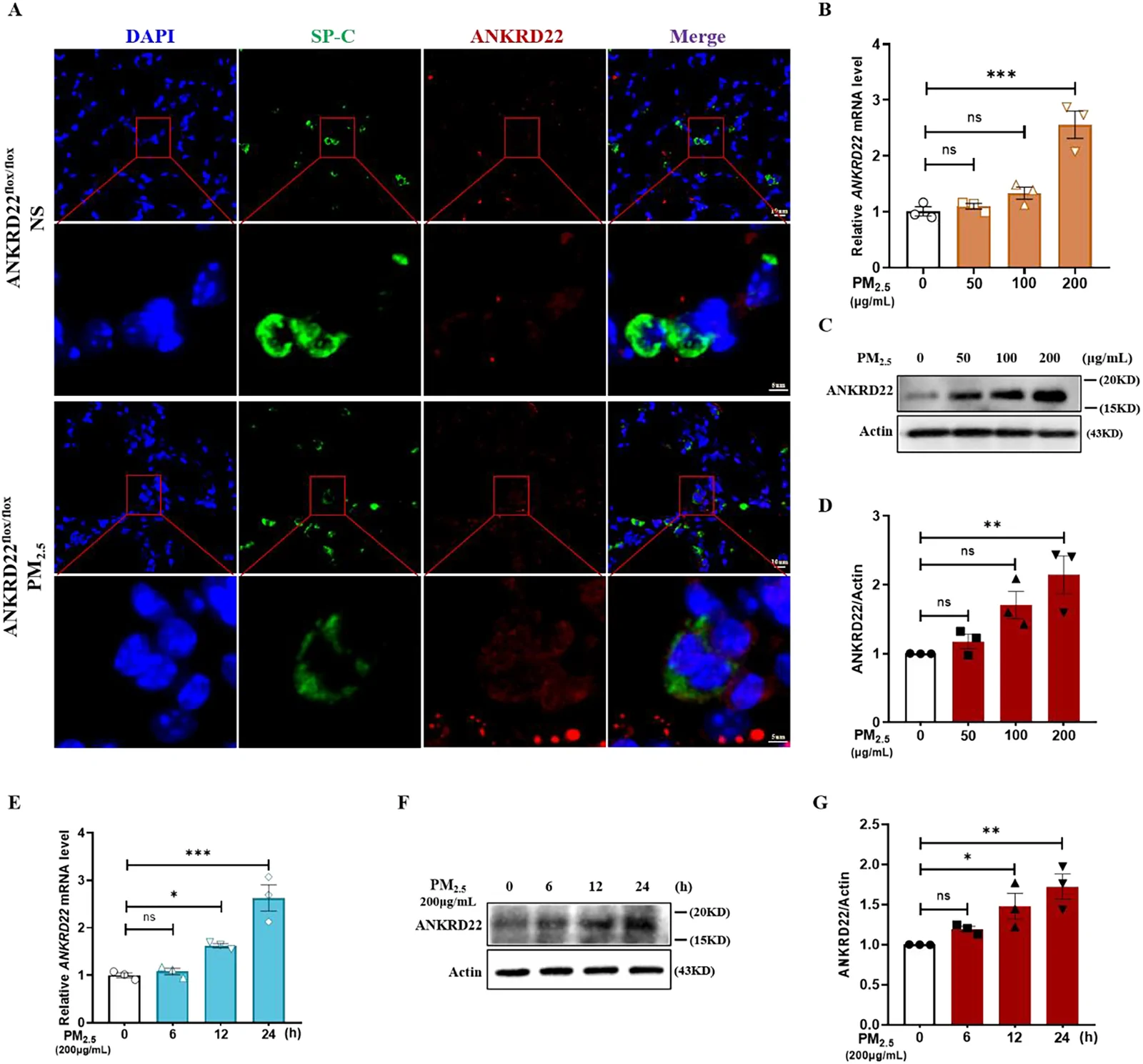
PM_2.5_ upregulates ANKRD22 expression in AT-II cells *in vivo* and *in vitro*. **(A)** Representative immunofluorescence co-staining images and semi-quantitative analysis of SP-C antibody (green) and ANKRD22 antibody (red) in murine lung tissues. Scale bars: 10 μm (overview), 5 μm (inset). DAPI stained the nuclei; Cell-type marker: SP-C antibody (AT-II cell); Target protein: ANKRD22 antibody (intracellular ANKRD22); Merged images: DAPI + SP-C + ANKRD22 overlay. **(B–D)** Following 24 h exposure of A549 cells to PM_2.5_ at varying concentrations (0, 50, 100, and 200 μg/mL), ANKRD22 expression was analyzed by qRT-PCR for mRNA levels **(B)**, while Western blot assessed ANKRD22 protein expression **(C)** with semi-quantitative results shown **(D)**. **(E–G)** A549 cells were exposed to 200 μg/mL PM_2.5_ for 0, 6, 12, and 24 h, followed by qRT-PCR analysis of ANKRD22 mRNA expression **(E)**, ANKRD22 protein levels were assessed by Western blot **(F)** and semi-quantified **(G)**. Multiple comparisons were performed using one-way ANOVA followed by Bonferroni’s *post hoc* test. Experimental data analysis results are expressed as mean ± SEM. Data are representative of three biological replicates with similar results (n = 3 per group)**;** *P < 0.05, **P < 0.01, ***P < 0.001. ns, non-significant.

### 3.2 Generation of cell-ANKRD22 knockout mice targeting alveolar type II epithelial cells

To further explore the functional role of ANKRD22 in PM_2.5_-induced murine airway inflammation, we generated AT-II cell-specific ANKRD22 knockout mice (*Sftpc-MerCreMer-ANKRD22^flox/flox^*) using CRISPR/Cas9 technology, with *ANKRD22^flox/flox^* mice serving as controls. The specific breeding strategy is illustrated in **Figure 2A**. Both *ANKRD22^flox/+^* mice and *Sftpc-MerCreMer* transgenic mice (which specifically express Cre recombinase in AT-II cells) were obtained from Cyagen Biosciences. These transgenic mice were intercrossed to generate the desired genotypes: *Sftpc-MerCreMer-ANKRD22^flox/flox^* mice and their control *ANKRD22^flox/flox^* counterparts (**Figure 2B**). The targeting construct was designed with loxP sites flanking exons 4–6 of the ANKRD22 gene, located on chromosome 19. Upon tamoxifen induction (0.2 mg/g body weight), Cre recombinase-mediated excision of exons 4–6 resulted in specific knockout of ANKRD22 in AT-II cells (designated as *Sftpc-MerCreMer-ANKRD22^flox/flox^*-Tamo+, abbreviated as AT-II-*ANKRD22^△/△^*). The knockout efficiency was confirmed by immunofluorescence co-staining of SP-C (green) and ANKRD22 (red) in lung tissue sections (**Figure 2C**). Results showed efficient deletion of ANKRD22 specifically within AT-II cells following tamoxifen induction.

**Figure 2:**
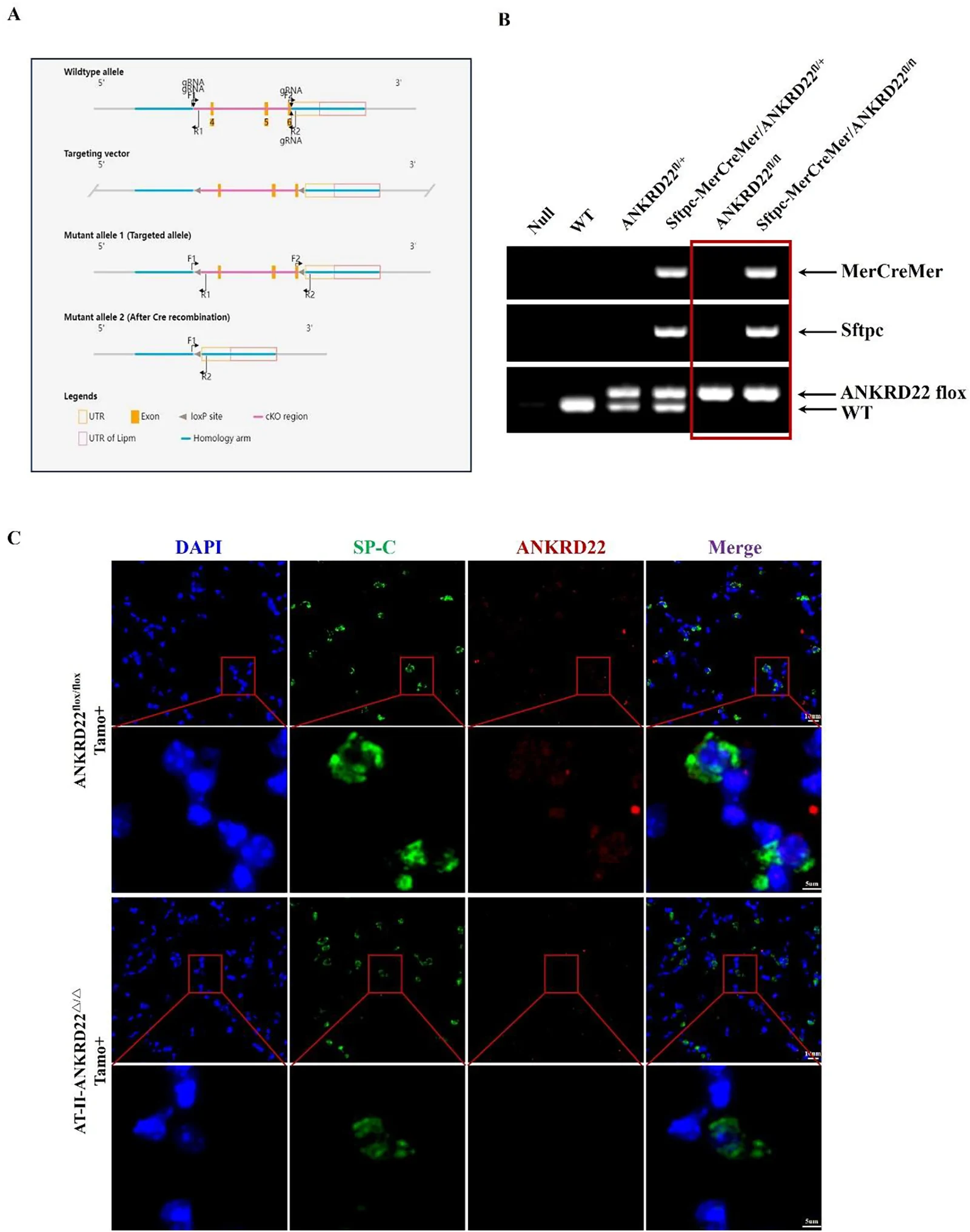
Generation of cell-ANKRD22 knockout mice targeting alveolar type II epithelial cells. **(A)** Schematic diagram of the strategy for generating alveolar type II epithelial cell-specific ANKRD22 knockout mice (*Sftpc-MerCreMer-ANKRD22^flox/flox^*) using CRISPR/Cas9 technology. **(B)** Representative genotyping results from the breeding process of transgenic mice. **(C)** AT-II-*ANKRD22^△/△^* mice and littermates (*ANKRD22^flox/flox^* mice) were received intraperitoneal injection of tamoxifen (0.2 mg/g) every other day for a total 4 injections, immunofluorescence co-staining of SP-C (green) and ANKRD22 (red) was performed on mouse lung sections. Scale bars: 10 μm (overview), 5 μm (inset). DAPI stained the nuclei; Cell-type marker: SP-C antibody (AT-II cell); Target protein: ANKRD22 antibody (intracellular ANKRD22); Merged images: DAPI + SP-C + ANKRD22 overlay. AT-II-*ANKRD22^△/△^* mice, AT-II cell-specific ANKRD22 knockout mice; Tamo, Tamoxifen.

### 3.3 ANKRD22 deficiency in murine AT-II cells protects against PM_2.5_-induced airway inflammation

Next, we investigated whether ANKRD22 in AT-II cells contributes to PM_2.5_-induced airway inflammation. To address this, we used tamoxifen inducible AT-II cell-specific *ANKRD22* knockout mice (*Sftpc-MerCreMer-ANKRD22^flox/flox^*), abbreviated as AT-II-ANKRD22*^△/△^*. Following tamoxifen pretreatment, both AT-II-*ANKRD22^△/△^* mice and their littermate controls (*ANKRD22^flox/flox^*) were exposed to PM_2.5_ via intratracheal instillation (**Figure 3A**). Efficient ANKRD22 ablation in AT-II cells was verified by immunofluorescence staining (**Figure 3B**).

**Figure 3:**
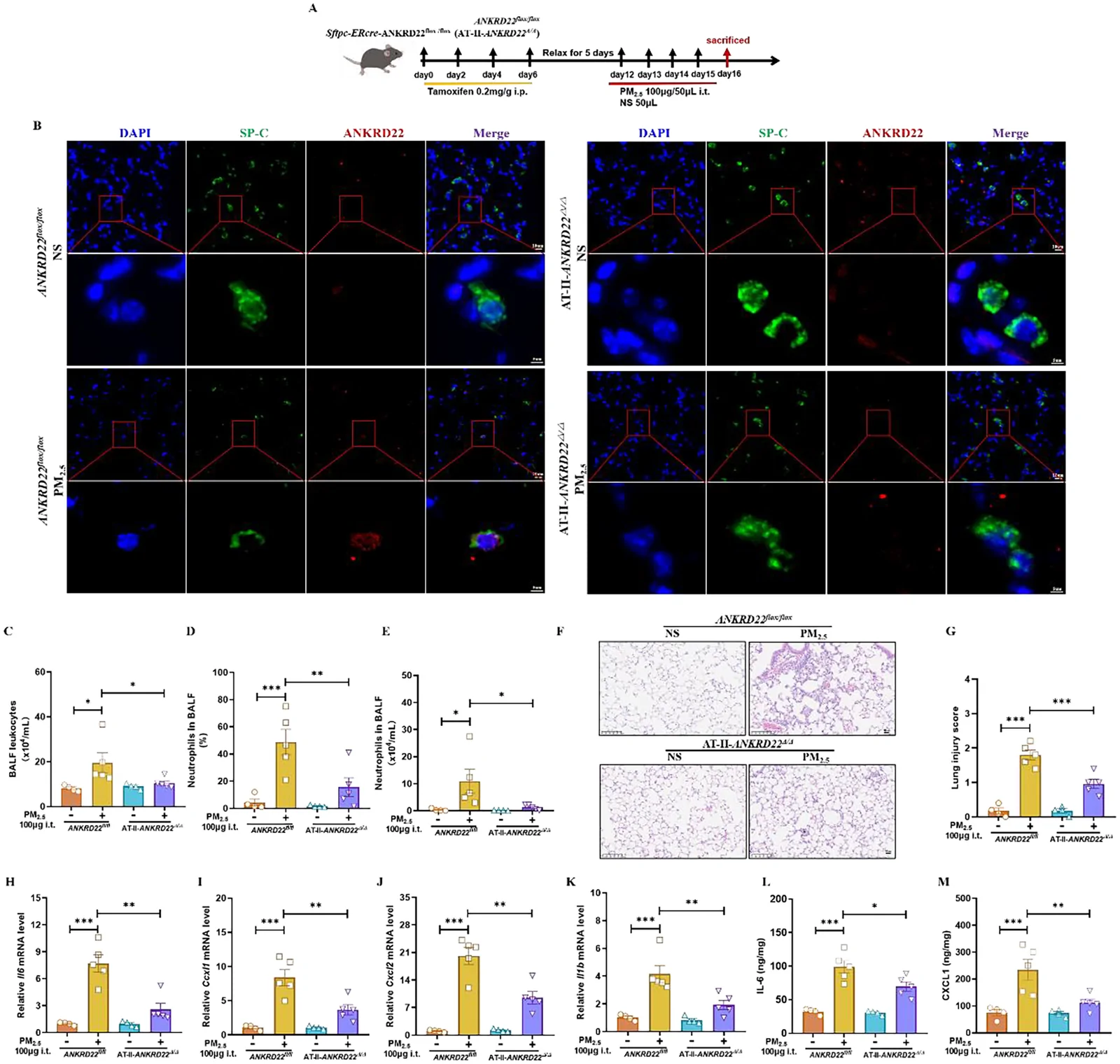
ANKRD22 deficiency in murine AT-II cells protected against PM_2.5_-induced airway inflammation. **(A)** Schematic diagram of PM_2.5_-induced airway inflammation model establishment in AT-II-*ANKRD22^△/△^* mice and *ANKRD22^flox/flox^* mice. **(B)** Representative immunofluorescence co-staining images of ANKRD22 (red) and SP-C (green) in paraffin-embedded lung tissue sections from mice. Scale bars: 10 μm (overview), 5 μm (inset). DAPI stained the nuclei; Cell-type marker: SP-C antibody (AT-II cell); Target protein: ANKRD22 antibody (intracellular ANKRD22); Merged images: DAPI + SP-C + ANKRD22 overlay. **(C–E)** BALF was collected, total leukocyte count **(C)**, neutrophil percentage **(D)** and neutrophil count **(E)** were calculated in BALF. **(F, G)** Representative HE-stained images of murine lung tissue sections were shown **(F)**, scale bar = 50 μm. Semi-quantitative analysis of HE staining in murine lung tissues was conducted **(G)**. **(H–K)** mRNA expression levels of *Il6* **(H)**, *Cxcl1* **(I)**, *Cxcl2* **(J)**, and *Il1b* **(K)** in murine lung tissues were detected by qRT-PCR. **(L, M)** ELISA detection of IL-6 **(L)** and CXCL1 **(M)** protein levels in mouse lung tissue homogenates. BALF differential cell counting and Histological scoring were performed blinded to group allocation. Multiple comparisons were performed using one-way ANOVA followed by Bonferroni’s *post hoc* test. Experimental data analysis results are expressed as mean ± SEM. Data are representative of three biological replicates with similar results (n = 4 mice in the control group of *ANKRD22^flox/flox^* and AT-II-*ANKRD22^△/△^* mice, n = 5 mice in the PM_2.5_-treated group of *ANKRD22^flox/flox^* and AT-II-*ANKRD22^△/△^* mice); *P < 0.05, **P < 0.01, ***P < 0.001.

PM_2.5_ exposure increased total leukocyte count, neutrophil percentage, and neutrophil numbers in BALF in control *ANKRD22^flox/flox^* mice, while these increases were substantially blunted in AT-II-*ANKRD22^△/△^* mice (**Figures 3C–E**). Consistent with this, HE staining showed that lung inflammation was notably alleviated in ANKRD22-deficient mice (**Figures 3F, G**). We next examined inflammatory gene expression and cytokine production in lung homogenates. qRT-PCR analysis showed that mRNA levels of *Il6*, *Cxcl1*, *Cxcl2*, and *Il1b* were markedly lower in AT-II- *ANKRD22^△/△^* mice than in *ANKRD22^flox/flox^* controls (**Figures 3H–K**). ELISA analysis further confirmed decreased protein levels of IL-6 and CXCL1 upon AT-II cell-specific *ANKRD22* deficiency (**Figures 3L, M**). Collectively, the *in vivo* experiments demonstrated that PM_2.5_-induced airway inflammation was substantially attenuated in mice with conditional ANKRD22 deletion restricted to AT-II cells.

### 3.4 ANKRD22 inhibition mitigates PM_2.5_-induced inflammation in A549 cells

*In vitro*, we transfected A549 cells with ANKRD22 siRNA or NC siRNA to further explore the importance of ANKRD22 in PM_2.5_-induced inflammation (**Figures 4A, B**). Among three independent siRNAs targeting ANKRD22, siRNA-640 showed the highest knockdown efficiency at both the mRNA and protein levels and was therefore selected for subsequent functional assays (**Supplementary Figures 1A, B**). In control siRNA-transfected cells, PM_2.5_ exposure significantly upregulated both mRNA and protein expression of ANKRD22. Knocking down ANKRD22, conversely, markedly reduced the mRNA expression of *IL6*, *IL8*, *IL1B*, *CXCL1*, and *CXCL2* (**Figures 4C–G**). Consistently, ELISA analysis of cell culture supernatants showed decreased IL-6 and IL-8 secretion in ANKRD22-knockdown A549 cells (**Figures 4H, I**). To exclude off-target effects, A549 cells were transfected with another independent siRNA, ANKRD22 siRNA-334, prior to PM_2.5_ stimulation. As shown in **Supplementary Figures 1C–E**, ANKRD22 silencing with siRNA-334 similarly attenuated PM2.5-induced upregulation of *IL6* and *IL8* mRNA. These *in vitro* findings suggest that genetic knockdown of ANKRD22 attenuates PM_2.5_-induced inflammatory responses in A549 cells.

**Figure 4:**
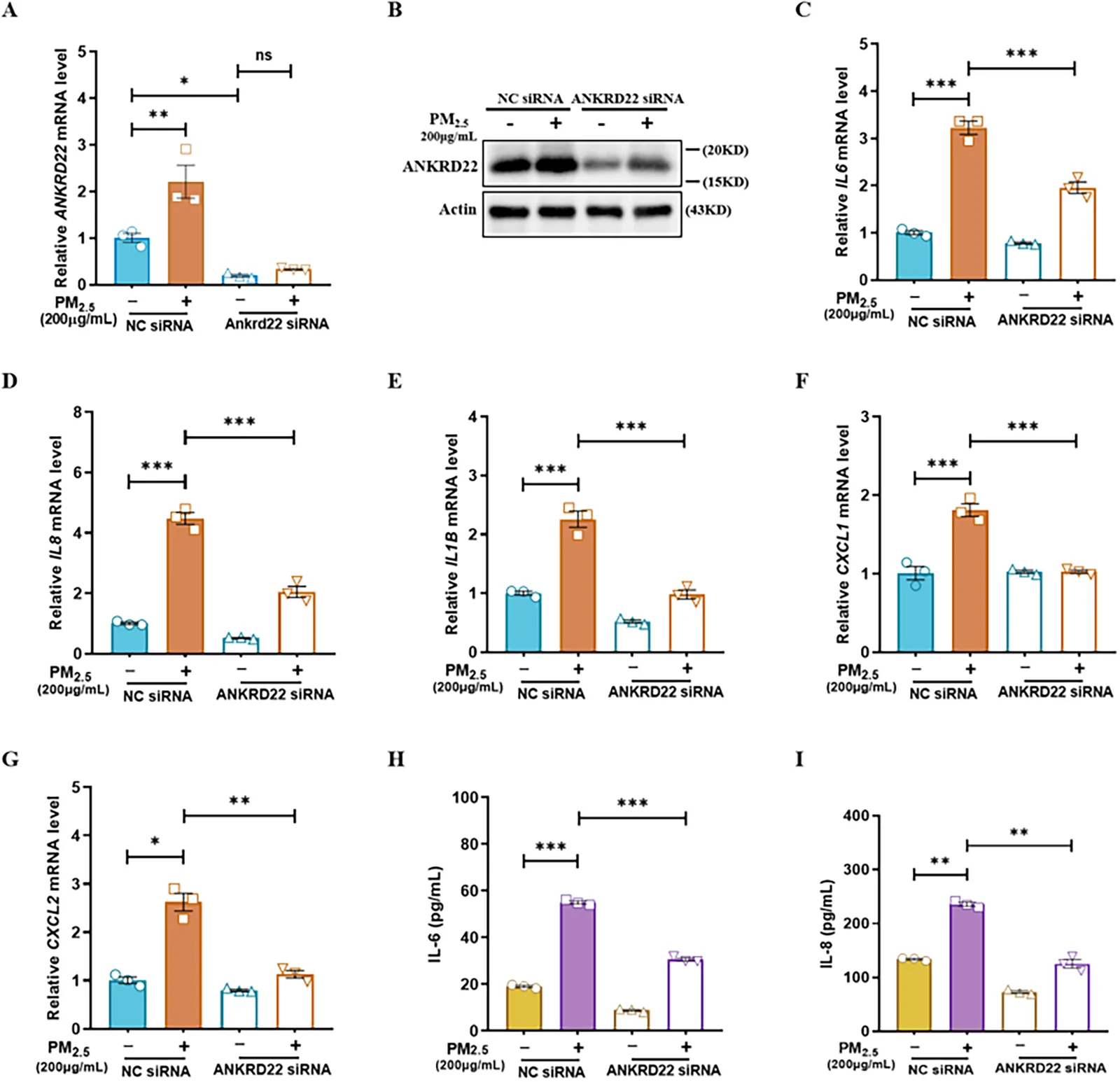
ANKRD22 inhibition mitigated PM_2.5_-induced inflammation in A549 cells. **(A, B)** A549 cells were transfected with ANKRD22 siRNA then stimulated with PM_2.5_, mRNA expression **(A)** and protein levels **(B)** of ANKRD22 were determined by qRT-PCR and Western blot. **(C–G)** mRNA expression levels of *IL6* **(C)**, *IL8* **(D)**, *IL1B* **(E)**, *CXCL1* **(F)**, and *CXCL2* **(G)** in A549 cells were detected by qRT-PCR. **(H, I)** ELISA measurement of IL-6 **(H)** and IL-8 **(I)** protein levels in A549 cells culture supernatants. Multiple comparisons were performed using one-way ANOVA followed by Bonferroni’s *post hoc* test. Experimental data analysis results are expressed as mean ± SEM. Data are representative of three biological replicates with similar results (n = 3 per group)**;** *P < 0.05, **P < 0.01, ***P < 0.001. ns, non-significant.

### 3.5 Genetic knockdown of ANKRD22 in A549 cells attenuates the activation of canonical and non-canonical NF-κB signaling pathways upon PM_2.5_ exposure

To further investigate the downstream signaling pathways associated with ANKRD22 in PM_2.5_-induced inflammation, we performed RNA-Seq analysis on A549 cells treated with ANKRD22 siRNA and PM_2.5_. The experiment included four groups (n = 3 per group): (1) NC siRNA + NS, (2) NC siRNA + PM_2.5_, (3) ANKRD22 siRNA + NS, and (4) ANKRD22 siRNA + PM_2.5_. DEGs were defined as |log_2_FC| ≥ 1 and an adjusted *P*-value < 0.05. PCA and Pearson correlation analyses confirmed the high reproducibility of the RNA-Seq dataset, with biological replicates clustering closely within each group and showing strong inter-sample correlations (**Supplementary Figures 2A, B**). Venn analysis identified 48 PM_2.5_-responsive DEGs that were reversed by ANKRD22 knockdown (**Figure 5A**), which were further visualized in a heatmap (**Figure 5B**). Subsequent KEGG and GO enrichment analyses of these DEGs (**Figures 5C, D**) highlighted significant enrichment in the NF-κB signaling pathway. To further evaluate pathway-level alterations, we performed GSEA, which revealed significant negative enrichment of the NF-κB signaling signature following ANKRD22 knockdown (**Figure 5E**), supporting transcriptome-wide suppression of NF-κB-associated inflammatory programs. Based on these findings, we further examined changes in NF-κB pathway-related proteins by western blotting (**Figure 5F**).

**Figure 5:**
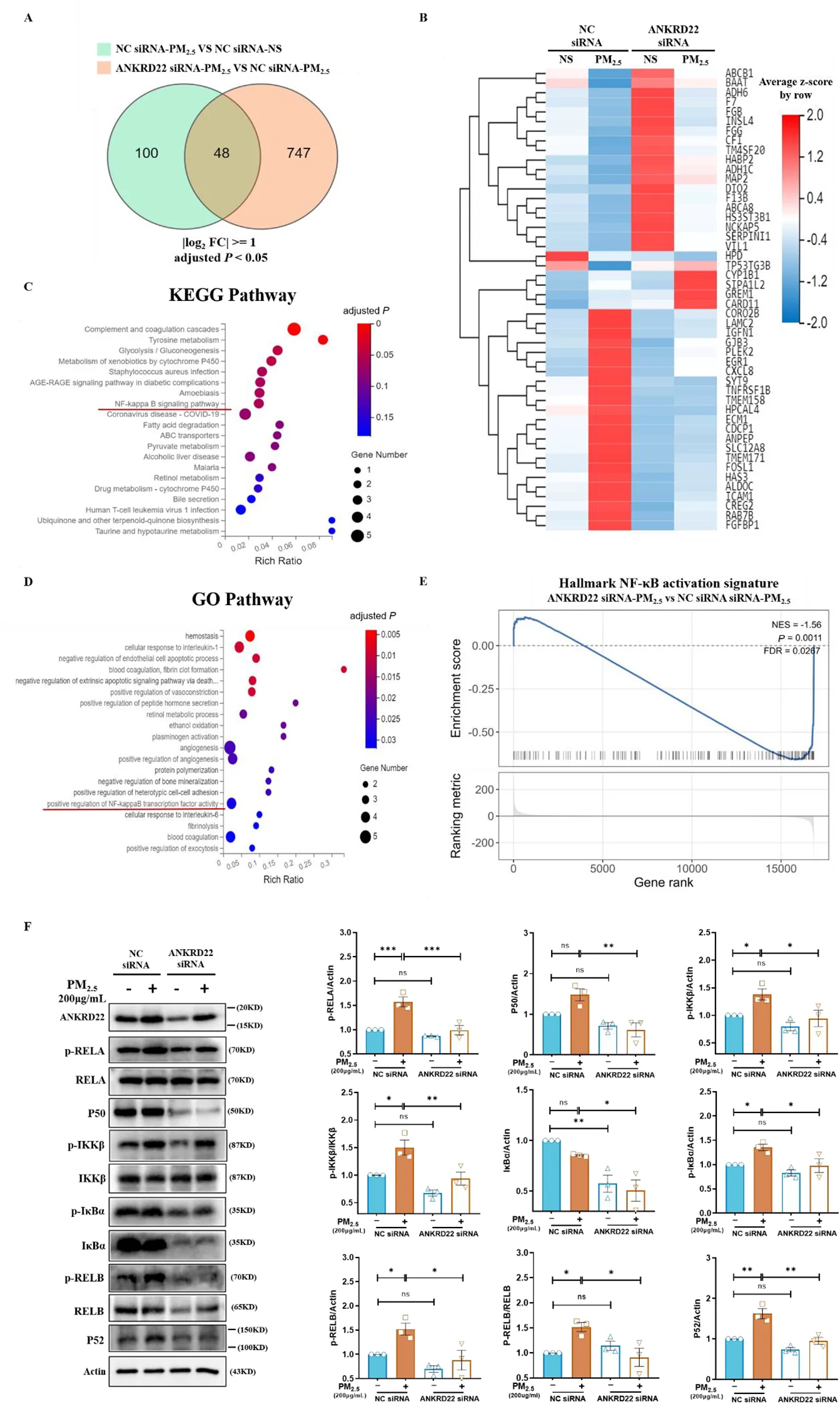
Genetic knockdown of ANKRD22 in A549 cells attenuates the activation of canonical and non-canonical NF-κB signaling pathways upon PM_2.5_ exposure. **(A)** Venn analysis of intersecting DEGs between NC siRNA-PM_2.5_ vs NC siRNA-NS comparison and ANKRD22 siRNA-PM_2.5_ vs NC siRNA-PM_2.5_ comparison. **(B)** Heatmap analysis of the 48 identified DEGs. **(C, D)** KEGG pathway enrichment analysis **(C)** and GO functional enrichment analysis **(D)** of the 48 DEGs. **(E)** GSEA of an NF-κB-associated gene signature following ANKRD22 knockdown in PM_2.5_-treated A549 cells. **(F)** WB analysis and semi-quantification of the protein expression and phosphorylation activation in the NF-κB signaling pathway in A549 cells after ANKRD2 siRNA transfection. DEGs, Differentially expressed genes; GSEA, Gene set enrichment analysis; FDA, False discovery rate. During RNA-Seq analysis, the thresholds for differential expression were set at |log_2_FC| ≥ 1 with an adjusted *P*-value< 0.05. Experimental data analysis results are expressed as mean ± SEM. Data are representative of three biological replicates with similar results (n = 3 per group); *P < 0.05, **P < 0.01, *** *P* < 0.001. ns, non-significant.

For the canonical NF-κB pathway, we found that PM_2.5_ exposure significantly enhanced phosphorylation of IKKβ and IκBα accompanied by reduced total protein levels of both molecules. Concurrently, we observed elevated P50 expression and enhanced RELA phosphorylation. Notably, ANKRD22 knockdown exerted opposing effects. We found the phosphorylated activation of IKKβ and IκBα was markedly reduced, accompanied by a significant reduction in IκBα protein levels. Moreover, suppression of both P50 expression and RELA phosphorylation was observed in ANKRD22-knockdown A549 cells upon PM_2.5_ exposure. We then turned to the non-canonical NF-κB pathway. Similarly, we found the protein expression of 52 and the RELB phosphorylation (p-RELB) were increased, resulting in an elevated p-RELB/RELB ratio. However, P52 expression and RELB phosphorylation were significantly inhibited upon ANKRD22 knockdown, suggesting a regulatory role of ANKRD22 in non-canonical NF-κB activation.

Taken together, genetic knockdown of ANKRD22 in A549 cells was associated with reduced activation of both canonical and non-canonical NF-κB signaling following PM_2.5_ exposure.

## 4 Discussion

A growing body of epidemiological and mechanistic evidence has established that exposure to ambient particulate matter (PM) is associated with adverse health outcomes affecting multiple organ systems, with PM_2.5_ being one of the most harmful components (25–28). Particularly, PM_2.5_ is critically involved in promoting the initiation and progression of airway inflammation (29–31). Numerous studies have demonstrated that AT-II cell plays a crucial role in PM_2.5_-induced airway inflammation (32, 33). Our research reveals that PM_2.5_ upregulates the expression of ANKRD22 in AT-II cells, and ANKRD22 exerts an important regulatory effect on PM_2.5_-induced airway inflammation. Recent studies have identified ANKRD22 as a key role in the regulation of inflammatory processes, exhibiting unique bidirectional regulatory properties (15, 22).

One study has revealed that in a murine gastritis model, ANKRD22 promotes the initiation and progression of gastritis by suppressing gastric mucosal repair and activating macrophages, with gastric epithelial cells and macrophages identified as the target cells (15). Conversely, in an imiquimod-induced psoriasis-like mouse model, ANKRD22-deficient mice exhibited significantly aggravated disease progression relative to wild-type controls. Further investigation demonstrated that ANKRD22 is predominantly expressed in dermal dendritic cells in this model, and its deficiency in these cells was identified as a critical driver of psoriasis exacerbation (22). Collectively, these findings suggest that ANKRD22 may function as a novel pro-resolution molecule in inflammation and could serve as a potential therapeutic target for psoriasis. However, reports on the role of ANKRD22 in respiratory diseases remain scarce to date. In a clinical study, Zhao et al. (17) reported that ANKRD22 expression was significantly elevated in non-small cell lung cancer (NSCLC) tumor tissues relative to adjacent non-cancerous counterparts. High ANKRD22 expression was strongly correlated with disease progression and reduced overall survival in NSCLC patients. Furthermore, *in vivo* mouse models and *in vitro* cellular experiments demonstrated that ANKRD22 is overexpressed in NSCLC and promotes tumor cell proliferation by upregulating the transcription factor E2F1, thereby accelerating NSCLC progression. This study provided the first evidence implicating ANKRD22 in respiratory diseases. Zhou et al. demonstrated that ANKRD22 is highly expressed in lung adenocarcinoma and facilitates tumor progression by enhancing glycolysis and promoting M2 polarization of tumor-associated macrophages (18). Separately, He et al. (34) identified ANKRD22 through GEO database screening, further statistical analyses and modeling revealed its association with inflammatory cell infiltration in pulmonary tuberculosis lesions, suggesting its potential utility as a biomarker for predicting the conversion from latent to active tuberculosis. However, the role of ANKRD22 in airway inflammatory disorders remains entirely unexplored.

For the first time, our study reveals that ANKRD22 expression is upregulated in AT-II cells in a PM_2.5_-induced airway inflammation model. Furthermore, PM_2.5_ exposure significantly drives ANKRD22 upregulation in A549 cells, in a manner dependent on both dose and time—higher concentrations and longer exposure durations yielded progressively greater increases in expression. Using AT-II cell-specific ANKRD22 knockout mice subjected to PM_2.5_ intratracheal instillation to establish an airway inflammation model, we demonstrated that ANKRD22 deletion significantly alleviated PM_2.5_-induced airway inflammation. Consistent with this *in vivo* observation, siRNA-mediated knockdown of ANKRD22 in A549 cells similarly attenuated PM2.5-triggered inflammatory responses. Of note, although ANKRD22 has been reported to exert context-dependent, and at times opposing, immunomodulatory effects across different disease settings, our data establish that AT-II cell-specific ablation of ANKRD22 effectively mitigates PM2.5-driven airway inflammation—unambiguously positioning ANKRD22 as a pro-inflammatory mediator in this specific pathological context. Previous study (15) in an EtOH/HCl-induced murine gastritis model demonstrated that gastric epithelial ANKRD22 inhibits mucosal repair by activating non-canonical Wnt signaling while suppressing the canonical Wnt pathway. Additionally, ANKRD22 promotes macrophage activation, exacerbating gastric mucosal injury. In LPS-stimulated primary mouse peritoneal macrophages, ANKRD22 knockout attenuated macrophage activation by inhibiting mitochondrial calcium influx and cytoplasmic NFAT (nuclear factor of activated T-cells) activation, consequently ameliorating gastric inflammation. In a psoriasis-like mouse model (22), ANKRD22 was found to directly bind NIK during non-canonical NF-κB activation, inducing its ubiquitination and degradation. Conversely, ANKRD22 deficiency led to sustained NIK accumulation, promoting p52/RELB nuclear translocation and subsequent IL-23 overproduction, which exacerbated psoriatic progression. Taken together, these findings collectively demonstrate that the regulatory functions of ANKRD22 are context-dependent, varying significantly across disease models and target cell types.

As described above, gastric epithelial ANKRD22 contributes to gastritis pathogenesis by modulating both canonical and non-canonical Wnt signaling pathways (15). In dendritic cells, by contrast, ANKRD22 exerts its regulatory effects primarily through the non-canonical NF-κB pathway (22). In the present study, transcriptomic analyses, including KEGG, GO, and GSEA, consistently indicated an association between ANKRD22 deficiency and NF-κB-associated inflammatory responses. It is well established that PM_2.5_ promotes activation of the canonical NF-κB pathway in pulmonary epithelial cells and macrophages (24, 35). however, whether PM_2.5_ engages the non-canonical NF-κB arm in this context has received little attention. During inflammatory responses, these two NF-κB branches are known to act in a functionally complementary manner, jointly orchestrating immune homeostasis and inflammatory equilibrium (10, 36–38). When we examined the signaling events more closely, ANKRD22-knockdown A549 cells, relative to PM_2.5_-exposed controls, showed markedly reduced expression of p50, p52, and IκBα, alongside attenuated phosphorylation of p-RELA, p-IKKβ, p-IκBα, and p-RELB—yet IKKβ expression was increased. These findings suggest that AT-II cell ANKRD22 is associated with the modulation of both canonical and non-canonical NF-κB signaling pathways in the context of PM_2.5_-induced inflammation.

Nevertheless, several limitations of this study should be acknowledged. First, although we employed AT-II cell-specific ANKRD22 knockout mice to assess the effect of ANKRD22 on PM_2.5_-induced airway inflammation *in vivo*, we did not further examine the inflammatory responses specifically within AT-II cells, nor did we delineate the mechanistic pathways through which ANKRD22 acts in this context. The current *in vivo* data establish a functional link between AT-II-cell ANKRD22 and PM2.5-induced airway inflammation at the organ level, but do not resolve whether the protective effect of ANKRD22 deletion is cell-autonomous or involves crosstalk with other pulmonary cell types. These issues warrant clarification in future investigations. Furthermore, with respect to the *in vitro* mechanistic studies, while RNA-Seq together with protein expression and phosphorylation analyses of the NF-κB pathway suggested that ANKRD22 modulates this signaling cascade, the detailed regulatory mechanisms remain to be elucidated. In addition, although A549 cells have been extensively employed in PM2.5-related toxicological and inflammatory studies (33, 39, 40), we recognize that A549 cells, as a transformed cell line, cannot fully recapitulate the biology of primary alveolar epithelial cells. Therefore, future investigations using primary AT-II cells, advanced three-dimensional culture systems, and other complementary cell models are warranted to confirm the generalizability and physiological relevance of our findings. Furthermore, the model was established by intratracheal instillation of PM_2.5_ in mice—a methodology that, despite being widely adopted in many high-quality reports, is not without constraints. Unlike natural inhalation exposure, intratracheal instillation involves differences in particle deposition patterns, bolus versus gradual delivery, and the inability to mimic chronic low-dose accumulation over extended periods. Future studies using more physiologically relevant exposure models will further improve our understanding of the long-term effects of PM_2.5_ exposure.

Current research on ANKRD22 in inflammatory diseases remains limited, particularly in respiratory disorders. Although our study reveals a pro-inflammatory role of AT-II cell-derived ANKRD22 in PM_2.5_-induced inflammation, further investigations are warranted to fully elucidate its regulatory mechanisms and biological functions. Oxidative stress is widely recognized as a key initiator of PM_2.5_-induced inflammatory responses (41, 42). Given that oxidative stress can activate NF-κB signaling (43), it is plausible that ANKRD22 may interact with or be regulated by oxidative stress signals. Future studies employing oxidative stress models, in combination with ANKRD22 gain- or loss-of-function approaches, will be necessary to clarify this potential crosstalk.

## 5 Conclusion

In conclusion, PM_2.5_-upregulated ANKRD22 in AT-II cells contributes to the development of airway inflammation, potentially involving both canonical and non-canonical NF-κB signaling pathways.

## Data Availability

The raw RNA-Seq data have been deposited in the Mendeley Data repository and are publicly available under the DOI: 10.17632/xgf3swc26z.1 (https://doi.org/10.17632/xgf3swc26z.1).

## Supplementary material

Supplementary Figure 1 Screening and validation of ANKRD22-targeting siRNA in A549 cells. **(A, B)** Three ANKRD22-targeting siRNAs (designated as 334, 459, and 640, respectively) were transfected into A549 cells, and knockdown efficiency was verified by qRT-PCR (mRNA level) and WB (protein level). **(C-E)** A549 cells were transfected with ANKRD22 siRNA-334 then stimulated with PM_2.5_, mRNA expression levels of *ANKRD22* **(C)**, *IL6* **(D)**, and *IL8* **(E)** in A549 cells were detected by qRT-PCR. Multiple comparisons were performed using one-way ANOVA followed by Bonferroni’s *post hoc* test. Experimental data analysis results are expressed as mean ± SEM. Data are representative of three biological replicates with similar results (n = 3 per group). ***P* < 0.01, ****P* < 0.001, ns, non-significant.

Supplementary Figure 2 PCA and Pearson correlation analyses of RNA-Seq. **(A)** Principal component analysis (PCA) showing separation of RNA-Seq samples among four experimental groups. **(B)** Pearson correlation heatmap of RNA-Seq samples based on VST-transformed expression profiles. Color indicates Pearson correlation coefficient (r).
