# Supplementary material for: ANKRD22 contributes to NF-κB-associated inflammatory responses in alveolar type II epithelial cells during PM2.5-triggered airway inflammation
Source: Front Immunol. 2026 Sep 16;17:1894350. doi: 10.3389/fimmu.2026.1894350 (PMC13623711; doi:10.3389/fimmu.2026.1894350)
Supplement: Supplementary file 1 [file DataSheet1.docx]

Supplementary Figure 1. **Screening and validation of ANKRD22-targeting siRNA in A549 cells.** (**A, B**) Three ANKRD22-targeting siRNAs (designated as 334, 459, and 640, respectively) were transfected into A549 cells, and knockdown efficiency was verified by qRT-PCR (mRNA level) and WB (protein level). (**C-E**) A549 cells were transfected with ANKRD22 siRNA-334 then stimulated with PM_2.5_, mRNA expression levels of *ANKRD22* (**C**), *IL6* (**D**), and *IL8* (**E**) in A549 cells were detected by qRT-PCR. Multiple comparisons were performed using one-way ANOVA followed by Bonferroni’s post hoc test. Experimental data analysis results are expressed as mean ± SEM. **Data are representative of three biological replicates with similar results (n = 3 per group)**. ***P* < 0.01, ****P* < 0.001, ns, non-signiﬁcant.

Supplementary Figure 2. PCA and Pearson correlation analyses of RNA-Seq. (A) Principal component analysis (PCA) showing separation of RNA-Seq samples among four experimental groups. (B) Pearson correlation heatmap of RNA-Seq samples based on VST-transformed expression profiles. Color indicates Pearson correlation coefficient (r).
